# *Trans*-Dominant Inhibition of Prion Propagation *In Vitro* Is Not Mediated by an Accessory Cofactor

**DOI:** 10.1371/journal.ppat.1000535

**Published:** 2009-07-31

**Authors:** James C. Geoghegan, Michael B. Miller, Aimee H. Kwak, Brent T. Harris, Surachai Supattapone

**Affiliations:** 1 Department of Biochemistry, Dartmouth Medical School, Hanover, New Hampshire, United States of America; 2 Department of Pathology, Dartmouth Medical School, Hanover, New Hampshire, United States of America; 3 Department of Medicine, Dartmouth Medical School, Hanover, New Hampshire, United States of America; University of Edinburgh, United Kingdom

## Abstract

Previous studies identified prion protein (PrP) mutants which act as dominant negative inhibitors of prion formation through a mechanism hypothesized to require an unidentified species-specific cofactor termed protein X. To study the mechanism of dominant negative inhibition *in vitro*, we used recombinant PrP^C^ molecules expressed in Chinese hamster ovary cells as substrates in serial protein misfolding cyclic amplification (sPMCA) reactions. Bioassays confirmed that the products of these reactions are infectious. Using this system, we find that: (1) *trans*-dominant inhibition can be dissociated from conversion activity, (2) dominant-negative inhibition of prion formation can be reconstituted *in vitro* using only purified substrates, even when wild type (WT) PrP^C^ is pre-incubated with poly(A) RNA and PrP^Sc^ template, and (3) Q172R is the only hamster PrP mutant tested that fails to convert into PrP^Sc^ and that can dominantly inhibit conversion of WT PrP at sub-stoichiometric levels. These results refute the hypothesis that protein X is required to mediate dominant inhibition of prion propagation, and suggest that PrP molecules compete for binding to a nascent seeding site on newly formed PrP^Sc^ molecules, most likely through an epitope containing residue 172.

## Author Summary

Over the past two decades, various investigators have observed that heterozygous animals possessing two different forms of the gene encoding the prion protein (PrP) are more difficult to infect with some strains of infectious prions than homozygous animals possessing only the most commonly occurring form of the gene encoding PrP for that species. In 1995, it was hypothesized that the inhibition of prion infection in heterozygous animals might be caused by competition between the two different types of PrP molecules for binding to a common cofactor required for prion propagation, provisionally named “protein X,” through a specific portion of the PrP molecule. Here, we report that mixing different purified PrP molecules together in test tube reactions lacking accessory proteins can also interfere with prion propagation. We also found that some mutations of the putative protein X binding site do not inhibit the formation of hamster prions in chemical reactions. Our work suggests that different PrP molecules most likely compete for binding to newly formed prions rather than an accessory protein cofactor, and argues against the existence of protein X.

## Introduction

Prion diseases are fatal neurodegenerative diseases with inherited, sporadic, and infectious etiologies [1]–[3]. The fundamental pathogenic event underlying prion diseases is generally believed to be the misfolding of the normal, host-encoded cellular prion protein (PrP^C^) into a pathogenic conformer (PrP^Sc^) [4], although in some experiments discordances between PrP^Sc^ levels and prion titers have been documented [5],[6]. Mature PrP^C^ is a ∼208 amino acid protein with a glycophosphatidyl inositol (GPI) anchor, two N-linked carbohydrate groups, and a single disulfide bond [7]–[10]. Experimentally, infectious prions can be formed *de novo* from a minimal set of components (PrP, lipid, and polyanionic molecules), which appear to form a high affinity physical complex [11],[12]. However, the precise mechanism by which PrP^Sc^ is formed from the conformational conversion of PrP^C^ has yet to be elucidated.

Studies examining the transmission of prions to transgenic mice expressing human or mouse/human chimeric PrP led to the hypothesis that a species-specific cofactor, termed protein X, is required for PrP^Sc^ formation [13]. Utilizing a cell culture model of prion formation, mouse (Mo) PrP single-point mutants that could not undergo conformational conversion to form PrP^Sc^ were identified [14]–[16]. These MoPrP mutants also acted in a dominant negative manner in that they prevented the conversion of wild type PrP^C^ when co-expressed in scrapie-infected cells. Two of the residues identified as conferring this dominant negative property correspond to naturally occurring polymorphic PrP variants. Sheep expressing Q171R PrP and humans expressing E219K PrP are both relatively resistant to prion infection [17]–[19], although cases of prion disease have been reported in animals with these genotypes [20]–[23]. In addition, substitution mutations to basic amino acids at residues 171 and 214 in MoPrP also yield dominant negative properties [14],[16]. In mouse PrP^C^, these four residues, 167 (homologous to sheep PrP residue 171), 171, 214, and 218, form a discontinuous epitope [24],[25], which was proposed to bind the protein X cofactor [14]. Hamster PrP^C^ harbors a homologous putative binding site [26],[27], and transgenic mice expressing mouse and hamster PrP^C^ molecules simultaneously are able to propagate both mouse and hamster prions [28]. Pharmacological studies demonstrated that compounds designed to bind to the putative protein X inhibit PrP^Sc^ formation in scrapie-infected neuroblastoma cells [29]. However, the protein X molecule has never been identified, and a recent study showed that Q218K PrP molecules reduced the rate of polymerization of wild type PrP molecules in a mixed polymerization reaction containing bacterially expressed PrP substrates but no additional cofactors [30]. Additionally, it has been shown that other heterologous PrP molecules lacking mutations of the putative protein X binding site can also interfere with conversion of MoPrP^C^ to MoPrP^Sc^ in cell culture and biochemical assays [31]–[33]. More broadly, the incubation periods of prion diseases appear to be controlled by a complex and poorly understood relationship between *Prn-p*, the gene encoding PrP, and prion strain [34],[35]. For instance, in mice, some prion strains have shorter incubation periods in mice expressing only the *Prn-p(a)* polymorphic allele than in mice expressing only the *Prn-p(b)* allele, while other strains cause disease faster in homozygous *Prn-p(b/b)* mice [36],[37]. Additionally, some strains produce longer incubation periods in *Prn-p(a/b)* heterozygote mice than in either homozygote [36]. In another example, sheep with Q171R (a putative mutation of the protein X binding site) mutant PrP alleles, previously thought to be resistant to prion infection, are susceptible to bovine spongiform encephalopathy as well as classical scrapie after long incubation periods [20]–[23]. Within this context, it should be noted that the bioassay experiments which originally supported the requirement for protein X in prion propagation were performed with a limited number of prion strains [13].

In this study, we use a combination of recently developed techniques to investigate the mechanism of dominant negative inhibition of prion formation *in vitro*. Specifically, we employ a Chinese Hamster Ovary (CHO) cell stable expression system to produce post-translationally processed PrP mutants, which can be used as substrates for the serial protein misfolding cyclic amplification (sPMCA) technique employing indirect sonication [38] in reconstituted [39] and purified systems [11]. The sPMCA technique has been used to generate infectious prions *in vitro* from both crude brain homogenate and purified PrP^C^ preparations [11],[38],[40]. Moreover, sPMCA experiments have shown that polyanions can facilitate the formation of infectious prions *de novo* in a completely prion-free environment [11]. In the present study, we used sPMCA to convert CHO-expressed PrP^C^ substrate into autocatalytic PrPres molecules, which were infectious to wild type animals. We then tested whether dominant negative MoPrP mutants and corresponding hamster (Ha) PrP mutants retain their dominant negative properties *in vitro*. Surprisingly, we found that a subset of hamster PrP mutants (T215K and Q219K) could be converted into autocatalytic PrPres products *in vitro*. These mutant PrP molecules also acted as *trans*-dominant inhibitors of both CHO-expressed and brain-derived wild type PrP^C^ conversion in a dose-dependent manner. Additionally, we found that pre-incubating wild type HaPrP^C^ substrate with either the scrapie template or a polyanionic cofactor, which is required for conversion in our purified system, did not prevent the inhibition of conversion by the dominant negative mutant Q172R HaPrP. Interestingly, T215K and Q219K HaPrPres could act as a seed to convert wild type HaPrP^C^, which indicates that PrP mutants physically interact with wild type PrP^C^. These results support a model in which dominant negative PrP mutants inhibit prion formation through direct interaction with a nascent seeding site on newly formed PrP^Sc^ molecules as opposed to interaction with a cellular cofactor such as protein X or with PrP^C^ molecules.

## Materials and Methods

### Animal welfare

All animals were handled in strict accordance with good animal practice as defined by the relevant national and/or local animal welfare bodies, and the Dartmouth College IACUC committee approved all animal work.

### Generation of CHO PrP cell lines

Each CHO cell line stably expressing wild type or mutant PrP was made separately by transfecting Flp-In™ CHO cells with pcDNA5/FRT/PrP plasmids using Lipofectamine™ 2000. Transfection and selection of polyclonal Flp-In™ CHO cell lines were carried out according to the manufacturer's protocol. After selection in hygromycin B, expression of PrP in each polyclonal cell line was confirmed by Western blot analysis. Cells were maintained at 37°C, 5% CO_2_, in Ham's F12 media containing 1% penicillin/streptomycin, 2 mM L-glutamine, and 10% fetal bovine serum. The correct identity of all stably transfected, PrP-expressing CHO cell lines used in this study was confirmed by DNA extraction and sequencing.

### Preparation of brain-derived and CHO-expressed PrP substrates

All procedures were performed at 4°C. Brain-derived HaPrP^C^ and CHO-expressed Q172R HaPrP was purified from hamster brains as previously described except, after immunopurification, the Q172R HaPrP was purified on a cobalt affinity column as described below instead of an SP cation exchange column [11]. To partially purify CHO-expressed PrP, each CHO cell line stably expressing PrP was grown to confluence in 10×150 mm dishes (Corning). Cells were rinsed with ice-cold PBS and harvested on ice with 10 ml lysis buffer [20 mM MOPS pH 7.0, 150 mM NaCl, 1% Triton-X 100, 1% DOC plus Complete™ EDTA-free protease inhibitors (Roche)]. The lysate was Dounce homogenized and allowed to solubilize on ice for 30 min. The solubilized lysate was then subjected to ultracentrifugation at 100,000×*g* for 1 hr at 4°C and the supernatant was passed through a 0.2 µm syringe filter (Millipore). The filtered supernatant was applied to a 2 ml IMAC-CoCl_2_ column (GE Healthcare, Piscataway, NJ). The column was washed with 10 ml of wash buffer [10 mM imidazole (in MOPS pH 7.0, 150 mM NaCl), 1% Triton-X 100] and eluted with 4 ml elution buffer [20 mM MES pH 6.4, 150 mM imidazole (in MOPS pH 7.0, 150 mM NaCl), 150 mM NaCl, 1% Triton-X 100]. The eluted samples containing PrP were divided into aliquots and stored at −70°C.

### Preparation of PrP^Sc^ and PrP^0/0^ mouse brain homogenates

PrP^Sc^ was prepared separately from two hamster strains Sc237 and 139H and one mouse strain, RML. In order to generate a 10% crude homogenate, each scrapie-infected brain was homogenized in 10 volumes (w/v) of PBS and centrifuged at 200×*g* for 30 s. The supernatant fraction containing PrP^Sc^ was divided into aliquots and stored at −70°C. To generate a PrP^0/0^ brain homogenate, a PrP^0/0^ mouse was first subjected to intracardiac perfusion with PBS, 5 mM EDTA. The brain was removed and Dounce homogenized in 5 ml PBS plus Complete™ protease inhibitors in order to generate a final homogenate concentration of 10% w/v. The homogenate was centrifuged at 1000×*g* for 30 sec and the supernatant fraction was divided into aliquots and stored at −70°C.

### Serial protein misfolding cyclic amplification (sPMCA)

Serial PMCA propagation experiments, adapted from Castilla *et al.* [38], were performed as previously described [11], except using both semi-purified CHO-expressed PrP and purified brain-derived HaPrP^C^ substrates. All reactions containing MoPrP substrate were supplemented with 2.5% PrP^0/0^ brain homogenate. One hundred microliter reactions containing HaPrP substrate were either supplemented with 2.5% PrP^0/0^ brain homogenate or 20 µg/ml synthetic poly(A) RNA. In the experiments testing the ability of mutant PrP to inhibit wild type PrP^C^ propagation, equal volumes of each PrP preparation were combined in the reactions. In the control reactions containing only wild type PrP^C^, an equal volume of cobalt IMAC elution buffer [20 mM MES pH 6.4, 150 mM imidazole (in MOPS pH 7.0, 150 mM NaCl), 150 mM NaCl, 1% Triton-X 100] was combined with the PrP. In pre-incubation experiments, a master reaction mix containing wild type HaPrP^C^ was pre-incubated with poly(A) RNA for 20 min at 37°C, or Sc237 template seed for 30 min at 37°C, both with shaking at 750 rpm in an Eppendorf Thermomixer, prior to addition of the Q172R mutant HaPrP. All propagation reactions described above were seeded on the first round of propagation with 10 µl 0.1% crude scrapie-brain homogenates diluted in PBS plus 1% Triton-X 100. In cross-seeding experiments, wild type and mutant CHO-expressed PrP template seed was derived from the 5^th^ round of an sPMCA propagation reaction originally seeded with 10 µl 0.1% Sc237 scrapie-brain homogenate in the first round.

### Supplemental methods

Additional experimental details are provided in Text S1.

## Results

### sPMCA propagation and infectivity of CHO-expressed PrP molecules

**Figure 1:**
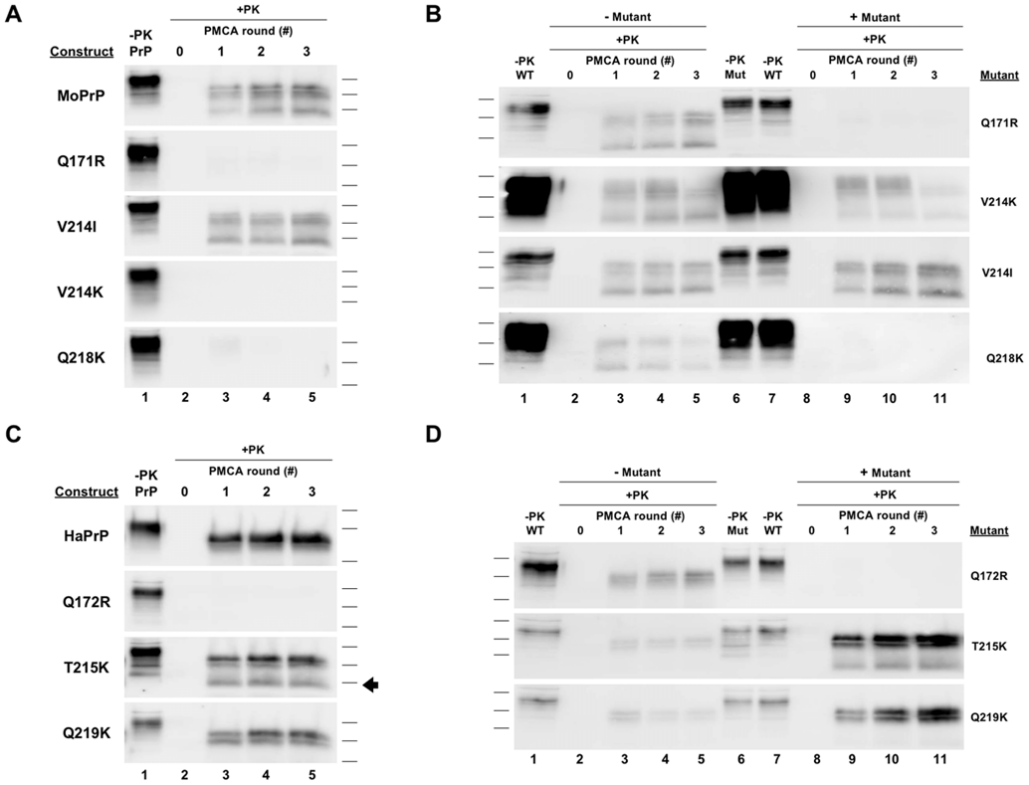
sPMCA propagation with CHO-expressed PrP substrates and Prnp^0/0^ brain homogenate. *(A)* Reactions containing either wild type or mutant mouse (Mo) PrP substrate alone were originally seeded with RML scrapie brain homogenate and propagated for three rounds of sPMCA. *(B)* Reactions containing either wild type MoPrP^C^ substrate alone (−*Mutant*, *lanes 2–5*) or in combination with either Q171R, V214I, V214K, or Q218K MoPrP mutant substrates at approximately equimolar concentrations (+*Mutant*, *lanes 8–11*), as indicated, were subjected to three rounds of serial propagation. *(C)* Reactions containing either wild type or mutant hamster (Ha) PrP substrate alone were originally seeded with Sc237 scrapie brain homogenate and propagated for three rounds of sPMCA reconstituted with Prnp^0/0^ brain homogenate. *(D)* Reactions containing either wild type HaPrP substrates alone (−*Mutant*, *lanes 2–5*) or in combination with either Q172R, T215K, or Q219K HaPrP mutant substrates at approximately equimolar concentrations (+*Mutant*, *lanes 8–11*), as indicated, were subjected to three rounds of serial propagation. An arrowhead demarks the ∼25 kDa PK-resistant T215K HaPrP species. All reactions were reconstituted with Prnp^0/0^ brain homogenate. In all blots, a sample containing wild type or mutant PrP substrate not subjected to proteinase K digestion is shown in the lanes preceding the corresponding PK-digested samples as a reference for comparison of electrophoretic mobility (*−PK PrP*, *WT, or Mut*). All other samples were subjected to limited proteolysis with proteinase K (25 µg/ml for 30 min. for mouse, 50 µg/ml for 1 hr. for hamster) at 37°C (*+PK*).

To study the properties of dominant negative mutant PrP molecules *in vitro* we began by expressing wild type mouse and hamster PrP^C^ in CHO cells, which do not express detectable levels of endogenous HaPrP^C^ protein [41]. Comparison of CHO-expressed PrP^C^ molecules to brain-derived PrP^C^ by SDS-PAGE revealed that the PrP^C^ expressed in CHO cells migrated between 26 and 43 kDa, a broader electrophoretic mobility pattern than brain-derived PrP^C^, which migrated between 26 kDa and ∼37 kDa (Figure S1, *lane 1* vs. *2–5* and *lane 6* vs. *7–10*). Deglycosylation of CHO-expressed MoPrP^C^ with peptide-N-glycosidase F (PNGase F) revealed that the increase in apparent molecular weight was due to more extensive glycosylation, as the core polypeptide of CHO-expressed and brain-derived MoPrP^C^ migrated equivalently on an SDS-PAGE gel (data not shown). We partially purified the mouse and hamster PrP^C^ from CHO cells by cobalt-affinity column chromatography for use as sPMCA substrates. We first conducted three-rounds of serial propagation reactions using CHO-expressed mouse PrP^C^ originally seeded with crude RML murine scrapie brain homogenate and reconstituted with PrP^0/0^ brain homogenate, which contains cofactors that facilitate the conversion of brain-derived HaPrP^C^ in sPMCA reactions [42] (Figure 1A). The results show that MoPrP^C^ was efficiently converted and propagated in this reaction, indicating that MoPrP^C^ expressed in CHO cells is a competent substrate for *in vitro* conversion into a protease-resistant and autocatalytic form of PrP (Figure 1A, *top blot*).

**Figure 2:**
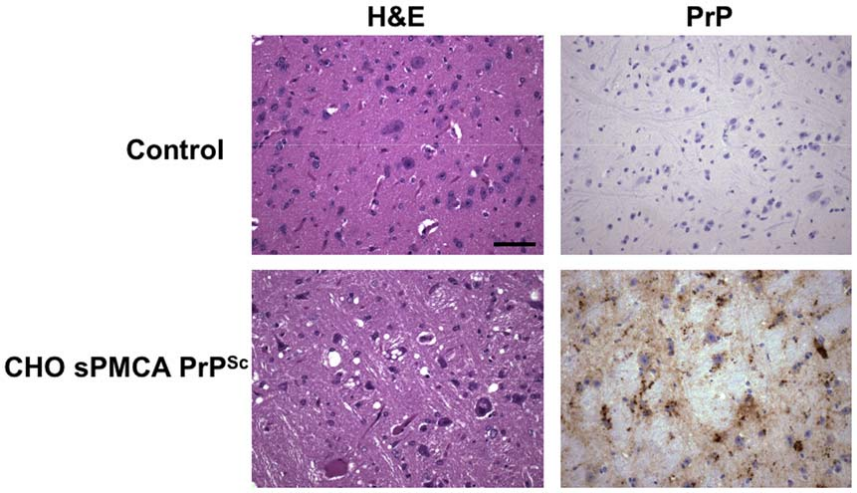
Neuropathology of hamsters infected with prions derived from CHO-expressed PrP substrate. Representative histological fields of the brainstem region in control animals and animals inoculated with CHO-expressed *in vitro*-generated PrP^Sc^ molecules. Top row: normal 166 day old Syrian golden hamster, mock inoculated with day 15 sPMCA propagation reaction containing the cobalt-purified fraction from untransfected CHO cells. Bottom row: terminally ill hamster inoculated with Sc237-seeded day 15 sPMCA reaction containing cobalt-prepared PrP^C^ expressed from CHO cells. Both reactions were seeded on day 0 with 0.01% Sc237-infected hamster brain homogenate. Hemotoxylin and eosin (H&E) and PrP immunohistochemical (3F4 antibody) staining are shown for each group. (Scale bar, 50 µm.)

We also conducted three-rounds of serial propagation reactions using the CHO-expressed hamster PrP^C^ substrate seeded with crude Sc237 hamster brain homogenate and supplemented with PrP^0/0^ brain homogenate (Figure 1C). The results show that HaPrP^C^ was efficiently converted and propagated in this reaction, indicating that HaPrP^C^ expressed in CHO cells is also a competent substrate for *in vitro* conversion into autocatalytic PrP (Figure 1C, *top blot*). In a separate reaction we carried out a seeded 15-round sPMCA propagation reaction with CHO-expressed wild type HaPrP^C^ substrate in order to generate a sample containing autocatalytic HaPrPres, which was beyond limiting dilution of the original scrapie template. To determine whether this HaPrPres was infectious, the sample from round 15 of the propagation reaction was intracerebrally inoculated into wild type hamsters. All of the inoculated hamsters (6/6) developed clinical scrapie with a mean incubation time of 141±17 days, whereas hamsters inoculated with a buffer control remain healthy to date (0/5) >380 days post-inoculation. The brains of hamsters inoculated with CHO-derived PrP^Sc^ displayed spongiform degeneration and PrP deposition consistent with the diagnosis of scrapie (Figure 2). The vacuolation and PrP deposition profiles of the hamsters inoculated with Sc237-seeded, CHO-derived PrP^Sc^ were very similar to hamsters inoculated with Sc237-seeded brain-derived PrP^Sc^ (Figure S2A and B).

### sPMCA propagation of mutant mouse PrP molecules reconstituted with brain homogenate

In order to test whether the MoPrP mutants Q171R, V214K, and Q218K retain dominant negative activity *in vitro*, we conducted serial protein-misfolding cyclic amplification (sPMCA) propagation assays using partially purified CHO-expressed MoPrP substrate molecules. These sPMCA reactions were seeded on day 0 with RML mouse prions, and reconstituted with Prnp^0/0^ brain homogenate in order to provide a source of potential brain chaperones, including the putative “protein X” (Figure 1A). Under these conditions, the Q171R, V214K, and Q218K MoPrP substrates all failed to undergo conversion, in contrast to the control wild type MoPrP substrate, which did form autocatalytic MoPrPres. Given these results, we were interested to know whether any of the negative MoPrP mutants could inhibit the *in vitro* conversion of wild type MoPrP^C^ in *trans*. To test this, we conducted seeded sPMCA propagation reactions containing both wild type and mutant MoPrP substrates (Figure 1B). As demonstrated in our previous experiment, wild type MoPrP^C^ alone is converted into autocatalytic MoPrPres during serial propagation (Figure 1B, *lanes 3–5*). When either the Q171R or Q218K MoPrP mutant was also added to the reaction, conversion of the wild type MoPrP^C^ substrate was completely abolished, indicating that both of these MoPrP mutants act as dominant negative substrates *in vitro* (Figure 1B, *lanes 9–11*, *top and bottom blots*). In contrast, the V214K MoPrP mutant, which did not act as a dominant inhibitor in ScN2a cells [14], also did not inhibit the conversion of wild type MoPrP^C^ (Figure 1B, *lanes 9–11*, *second blot*).

We also tested the MoPrP V214I mutant in parallel assays. Unlike the negative MoPrP mutants tested, V214I is capable of forming PrPres in ScN2a cells [14]. Correspondingly, we found that MoPrP V214I is an efficient substrate for RML-seeded sPMCA reactions (Figure 1A). Thus, the results for RML-seeded sPMCA propagation assays utilizing CHO-expressed substrates (Figure 1A and B) correlate completely with the previously published results of ScN2a assays for both positive and dominant negative MoPrP mutants tested [14].

### sPMCA propagation of mutant hamster PrP molecules reconstituted with brain homogenate

Previous studies of dominant negative PrP mutants in prion-susceptible cultured mouse cell lines were restricted to the mouse prion strains RML and 22L [14],[15],[43],[44]. To study the generalizability of these results and to examine the structure-function relationships of dominant negative PrP mutants in a different rodent species and prion strain, we utilized our CHO cell expression system and sPMCA to study these mutations in hamster PrP.

We were curious to know whether dominant negative properties of MoPrP mutants would be conferred to HaPrP with corresponding mutations. We began by generating CHO cell lines stably expressing the HaPrP mutants Q172R, T215K, and Q219K which correspond to the MoPrP dominant negative mutants Q171R, Q214K, and Q218K, respectively [14]. The SDS-PAGE electrophoretic mobility patterns of these HaPrP molecules were very similar to wild type HaPrP^C^ expressed in CHO cells (Figure S1, *lanes 7–10*). In general, we also observed that the HaPrP mutant T215K was enriched for the monoglycosylated and unglycosylated isoforms relative to the wild type, Q172R, and Q219K HaPrP constructs, though the degree of this glycoform difference varied between individual preparations (Figure S1, *lane 9* vs. *7*,*8*, and *10*).

To determine if the HaPrP mutants, Q172R, T215K, and Q219K, which correspond to the dominant negative MoPrP mutants Q171R, Q214K, and Q218K, respectively, could be converted into autocatalytic HaPrPres *in vitro* we conducted sPMCA propagation reactions containing mutant HaPrP substrate partially purified from CHO cells reconstituted with Prnp^0/0^ brain homogenate (Figure 1C, *bottom three blots*) [14]. Q172R HaPrP substrate failed to undergo conversion, in contrast to T215K and Q219K HaPrP mutants, which did form autocatalytic HaPrPres. Notably, unlike wild type and Q219K HaPrP substrates, the protease resistant pattern of the T215K HaPrP mutant included a ∼25 kDa species which is not formed during conversion of the other two constructs (Figure 1C, *arrowhead*).

As in our previous MoPrP experiments, we wanted to investigate if the HaPrP mutants Q172R, T215K, and Q219K could inhibit the conversion of wild type HaPrP. To test this, we conducted seeded sPMCA propagation reactions containing both wild type and mutant HaPrP substrates (Figure 1D). As demonstrated in our previous experiment, wild type HaPrP^C^ substrate alone was converted into autocatalytic HaPrPres in serial propagation reactions supplemented with Prnp^0/0^ brain homogenate (Figure 1C, *lanes 3–5*, *all blots*). Strikingly, when Q172R HaPrP substrate was added to the reaction, conversion of wild type HaPrP^C^ substrate was completely abolished, even though wild type HaPrP^C^ was present in molar excess (Figure 1D, *lanes 9–11*, *top blot*). In contrast, in reactions containing both wild type HaPrP^C^ substrate and either T215K or Q219K HaPrP substrate, autocatalytic HaPrPres was still formed (Figure 1D, *lanes 9–11*, *lower blots*). While these results do not indicate if T215K and Q219K HaPrP can inhibit the conversion of CHO-expressed wild type HaPrP^C^, we later found that these mutants can inhibit the conversion of brain-derived HaPrP^C^ (see below).

### sPMCA propagation of mutant PrP molecules stimulated by poly(A) RNA

A hypothesized mechanism for the properties of dominant negative PrP mutants is that they sequester protein X, a molecule proposed to be required for prion conversion [13],[14]. It was thought that mutations in these PrP molecules increase their affinity for binding protein X, relative to wild type PrP^C^. We sought to investigate this proposed mechanism by conducting prion propagation reactions using the sPMCA assay. To do this we carried out serial propagation reactions containing HaPrP substrate and poly(A) RNA instead of Prnp^0/0^ brain homogenate. We have previously demonstrated that sPMCA propagation reactions supplemented with synthetic homopolymeric poly(A) RNA can facilitate the formation of native hamster prions [11]. Using sPMCA, we were able to test whether dominant negative mutations in PrP could affect the ability of poly(A) RNA to stimulate *in vitro* conversion.

**Figure 3:**
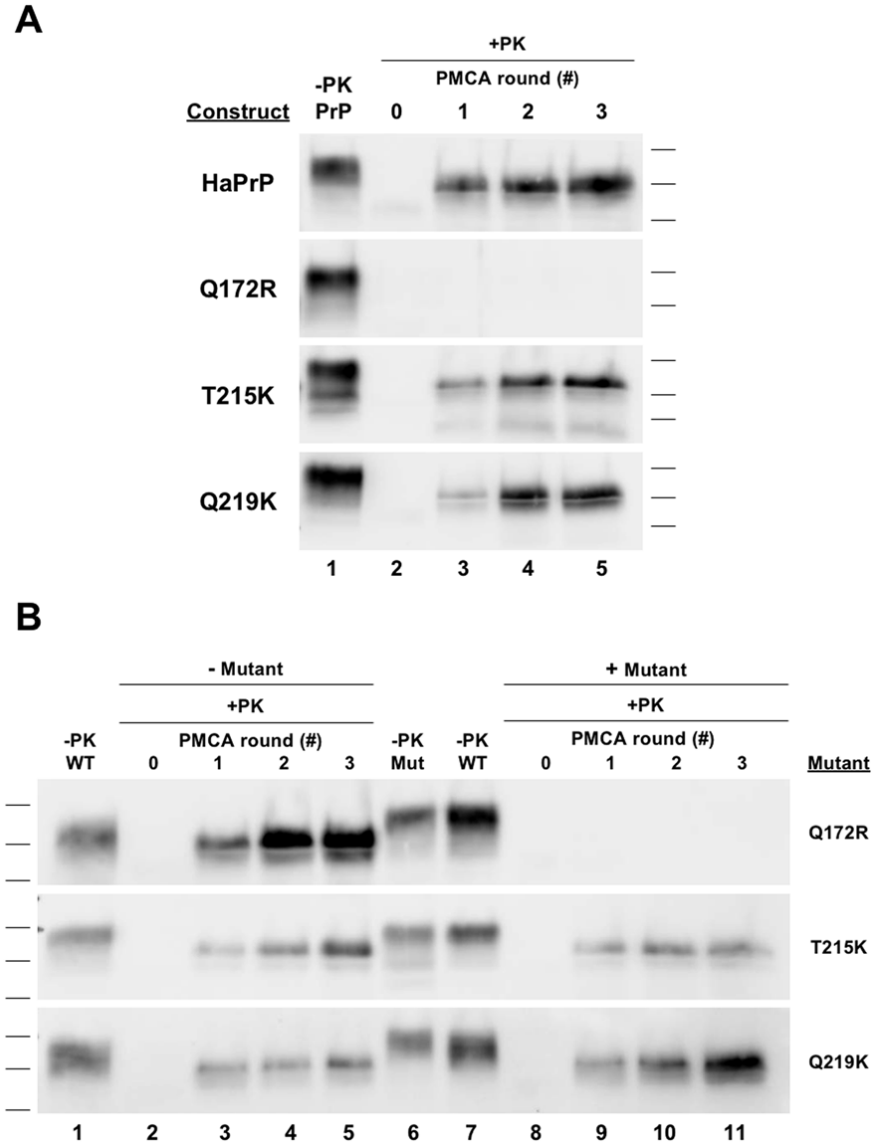
sPMCA propagation with Hamster PrP substrates and synthetic poly(A) RNA. *(A)* Reactions containing either wild type or mutant HaPrP substrate alone were originally seeded with Sc237 scrapie brain homogenate and propagated for three rounds of sPMCA. *(B)* Reactions containing either wild type HaPrP^C^ alone (−*Mutant*, *lanes 2–5*) or in combination with either Q172R, T215K, or Q219K HaPrP mutant substrates at ∼1∶2 (Mut∶WT) ratio (+*Mutant*, *lanes 8–11*), as indicated, were subjected to three rounds of serial propagation. All reactions were supplemented with synthetic poly(A) RNA. In all blots, a sample containing wild type or mutant HaPrP substrate not subjected to proteinase K digestion is shown in the lane(s) preceding the corresponding PK-digested samples as a reference for comparison of electrophoretic mobility (*−PK PrP, WT, or Mut*). All other samples were subjected to limited proteolysis with 50 µg/ml proteinase K for 1 hr at 37°C (*+PK*).

We first conducted three-rounds of serial propagation reactions seeded with crude Sc237 brain homogenate and supplemented with poly(A) RNA (Figure 3A). As in the reactions supplemented with brain homogenate, Q172R HaPrP substrate failed to undergo conversion, in contrast to T215K and Q219K HaPrP substrate, which formed autocatalytic HaPrPres (Figure 3A, *bottom three blots*). These results indicate that the inability of Q172R HaPrP substrate to undergo conversion is not due to a factor present in brain homogenate, unless that factor is mimicked by, or is itself poly(A) RNA.

We next examined whether Q172R, T215K, and Q219K HaPrP substrate could inhibit the conversion of wild type HaPrP^C^ substrate in reactions supplemented with poly(A) RNA. Again, as in reactions supplemented with brain homogenate, when sub-stoichiometric amounts of Q172R HaPrP substrate was added to the reaction, conversion of the wild type HaPrP^C^ substrate was abolished (Figure 3B, *lanes 9–11*, *top blot*).

As it is possible that the ability of the mutant HaPrP molecules to undergo *in vitro* conversion could be dependent upon, or affected by, the strain of scrapie used to seed the propagation reaction, we carried out similar reactions which were seeded with the 139H scrapie strain instead of the Sc237 strain (Figure S3). In this experiment we observed again that wild type HaPrP substrate was successfully propagated but Q172R HaPrP substrate failed to undergo conversion (Figure S3, *top two blots*). Likewise, T215K and Q219K HaPrP substrate propagated, though conversion was much less as compared to wild type HaPrP substrate conversion (Figure S3 *bottom blots vs. top*).

To test whether mutant PrP molecules might convert slowly into PrPres molecules, and whether mutant PrP might prolong the kinetics of WT PrPres formation, we performed sPMCA reactions in which we increased the duration of each individual propagation round. These experiments showed that purified Q172R HaPrP substrate did not form PrPres after 3×4 day propagation rounds in reactions supplemented with poly(A) RNA (Figure S4, *lanes 13–16, bottom blot*). Similar results were obtained with Q171R, V214K, and Q218K MoPrP substrates in sPMCA reactions supplemented with Prnp^0/0^ brain homogenate (data not shown). The results also indicate that the inhibition of WT HaPrPres formation by Q172R HaPrP was also unaffected by increasing the duration of the propagation rounds to 4 days (Figure S4, *lanes 8–11, bottom blot*).

### Biochemical characterization of mutant PrP molecules expressed in CHO cells

Given the unexpected results that the T215K HaPrP, and Q219K HaPrP mutants could form autocatalytic PrPres products in sPMCA reactions, we wanted to confirm that these results were not due to any abnormal cellular processing of the expressed PrP molecules. Native, brain-derived PrP^C^ is normally trafficked to the plasma membrane where it is attached to the outer leaflet *via* a GPI anchor [45]. To be certain our HaPrP expressed in CHO cells were properly trafficked to the surface of the cell, we treated each cell line with phosphatidylinositol-specific phospholipase C (PI-PLC), an enzyme which cleaves the linkage between the GPI and protein moieties of GPI-anchored proteins, releasing the protein into the extracellular environment. In cell lines not treated with PI-PLC, wild type and mutant HaPrP were detectable in CHO cell lysates with little to none detected in PI-PLC incubation media (Figure S5, *lane 1* vs. *2*), demonstrating that the expressed HaPrP molecules are normally associated with the CHO cells and not secreted into the extracellular environment. In contrast, upon treatment of the cell lines with PI-PLC, the majority of HaPrP was detected in the media and not in the cell lysate (Figure S5, *lanes 3* vs. *4*), indicating that the HaPrP expressed in these lines is attached to the outside of the plasma membrane as the GPI anchor was accessible to cleavage by PI-PLC. We previously reported that treatment of PrP^C^ with PI-PLC results in inefficient immunodetection of PrP^C^ bound to PVDF membrane [46]. This phenomenon likely explains the overall decreased detection of HaPrP in the cell lines treated with PI-PLC relative to the untreated cells (Figure S5, *lane 2* vs. *lanes 3–4* combined). These results show that, similar to native HaPrP^C^, CHO-expressed wild type and mutant HaPrP stably expressed in CHO cells are properly trafficked and attached to the outer leaflet of the plasma membrane.

We next tested whether the HaPrP expressed in our CHO cells was detergent soluble under non-denaturing conditions, a biochemical characteristic of brain-derived PrP^C^ [47] (Figure S6). CHO cells lines expressing wild type and mutant HaPrP were solubilized in detergent and subjected to ultracentrifugation in order to separate the soluble and insoluble fractions. The majority of each HaPrP construct was recovered in the detergent-soluble supernatant fraction with little or no HaPrP in the detergent-insoluble pellet fraction (Figure S6, *lane 2* vs. *lane 3*). Together these data indicate that HaPrP expressed in our CHO cell lines undergoes cellular processing and trafficking in a manner similar to native, brain-derived PrP^C^.

### Dose-dependent inhibition of brain-derived PrP^C^ substrate by mutant PrP molecules in *trans*

**Figure 4:**
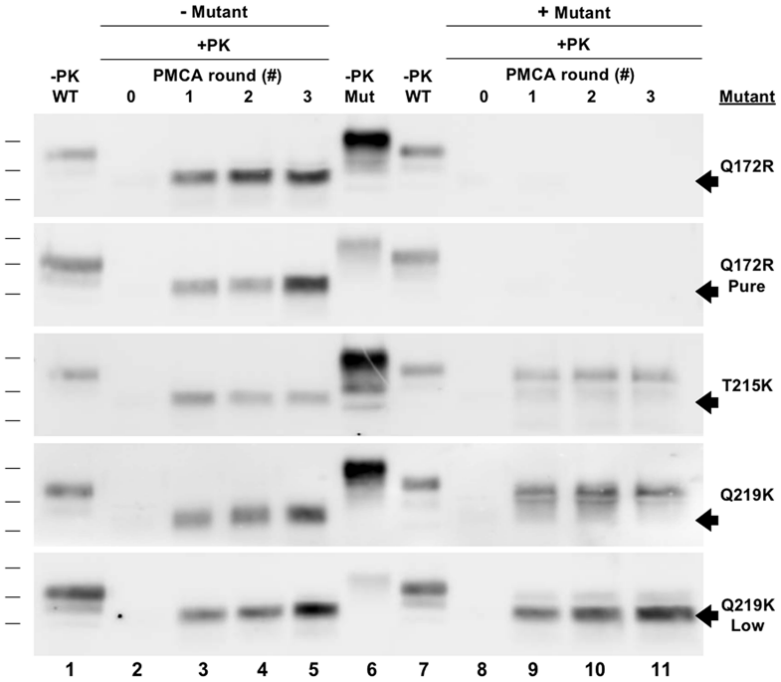
Inhibition of hamster brain PrP^C^ conversion by dominant negative mutant PrP. Reactions containing either purified brain-derived HaPrP^C^ alone (−*Mutant*, *lanes 2–5*) or in combination with either Q172R, immunopurified Q172R (*Q172R Pure*), T215K, or different concentrations of Q219K HaPrP mutant substrates (+*Mutant*, *lanes 8–11*), as indicated, were subjected to three rounds of serial propagation. Q172R Pure was tested at ∼1∶2 (Mut∶WT) ratio; Q172R Low was tested at ∼1∶5 ratio; and all other conditions were tested at ∼5∶1 ratio. In each blot, an arrowhead demarks the expected mobility of the ∼27–30 kDa PK-resistant brain-derived PrP^Sc^ species. All reactions were supplemented with synthetic poly(A) RNA. In all blots, a sample containing wild type or mutant HaPrP substrate not subjected to proteinase K digestion is shown in the lane(s) preceding the corresponding PK-digested samples as a reference for comparison of electrophoretic mobility (*−PK WT or Mut*). All other samples were subjected to limited proteolysis with 50 µg/ml proteinase K for 1 hr at 37°C (*+PK*).

While it was clear the Q172R HaPrP substrate could inhibit the conversion of wild type HaPrP^C^ substrate, we still could not conclude whether T215K and Q219K HaPrP substrates possess the same dominant negative properties. Our HaPrP substrate expressed in CHO cells has a higher relative molecular weight as compared to brain-derived HaPrP^C^; thus, the molecular weight of protease-resistant PrP derived from the conversion of these different PrP substrates should also be distinguishable by SDS-PAGE. We decided to exploit this biochemical difference between HaPrP substrates to determine if T215K and Q219K HaPrP substrate act as dominant negatives. We conducted seeded sPMCA propagation reactions containing both immunopurified brain-derived wild type HaPrP^C^ and mutant HaPrP substrates supplemented with poly(A) RNA (Figure 4). Consistent with previous studies, brain-derived wild type HaPrP^C^ substrate was converted into autocatalytic HaPrP^Sc^ in serial propagation reactions supplemented with poly(A) RNA (Figure 4, *lanes 3–5*, *all blots*). When Q172R HaPrP substrate was added to the reaction, conversion of brain-derived wild type HaPrP^C^ substrate was blocked (Figure 4, *lanes 9–11*, *top blot*).

In reactions containing both wild type HaPrP^C^ substrate and either the T215K or Q219K HaPrP substrate, autocatalytic HaPrPres was still formed (Figure 4, *lanes 9–11*, *lower middle blots*). However, the molecular weight of the HaPrPres formed in these reactions corresponded to the weight of T215K and Q219K HaPrPres, and not brain-derived HaPrP^Sc^ (predicted mobility shown with arrowheads). The protease resistant band corresponding to brain-derived HaPrP^Sc^ was not present in reactions containing both wild type HaPrP^C^ and mutant HaPrP substrates (Figure 4, *lanes 9–11 vs. 3–5*). Similar to Q172R, this finding shows T215K and Q219K HaPrP substrate can inhibit the conversion of brain-derived wild type HaPrP^C^ substrate and, therefore, act as dominant negatives *in vitro*.

As the concentration of the mutant HaPrP substrate in these reactions was greater than brain-derived HaPrP^C^ substrate, we wanted to test whether conversion could be inhibited at lower mutant PrP substrate concentrations. In order to investigate this we conducted a propagation reaction containing purified brain-derived HaPrP^C^ substrate and a sub-stoichiometric concentration of Q219K HaPrP substrate (*Q219K Low*) (Figure 4, *bottom blot*). Interestingly, under these conditions both wild type HaPrP^C^ and Q219K HaPrP substrates were converted into autocatalytic, protease-resistant HaPrP as indicated by two distinct bands (Figure 4, *lanes 9–11*, *bottom blot*). This finding demonstrates that inhibition of wild type PrP^C^ conversion by Q219K occurs in a dose-dependent manner; at stoichiometrically higher doses Q219K PrP inhibits conversion but at stoichiometrically lower doses, wild type PrP^C^ still occurs. Furthermore, in this case, *trans*-dominant interference appears to be asymmetrical; Q219K appears to block conversion of WT PrP (Figure 4, *lanes 9–11*, *4th blot from top*, see absence of band at the arrowhead), but not *vice versa* (Figure 4, *lanes 9–11*, *bottom blot*, see presence of band above the arrowhead).

### Dominant negative inhibition with purified PrP molecules

In order to exclude the possibility that an external molecule that co-purifies with mutant PrP substrate is required for dominant negative inhibition we conducted propagation reactions containing both purified brain-derived wild type HaPrP^C^ and purified Q172R (*Q172R Pure*) HaPrP substrate (Figure 4). As in the reactions containing partially purified Q172R HaPrP substrate, fully purified Q172R HaPrP substrate still maintained the ability to inhibit the conversion of purified HaPrP^C^ substrate (Figure 4, *lanes 9–11*).

### Pre-incubation of PrP with poly(A) RNA and scrapie template

**Figure 5:**
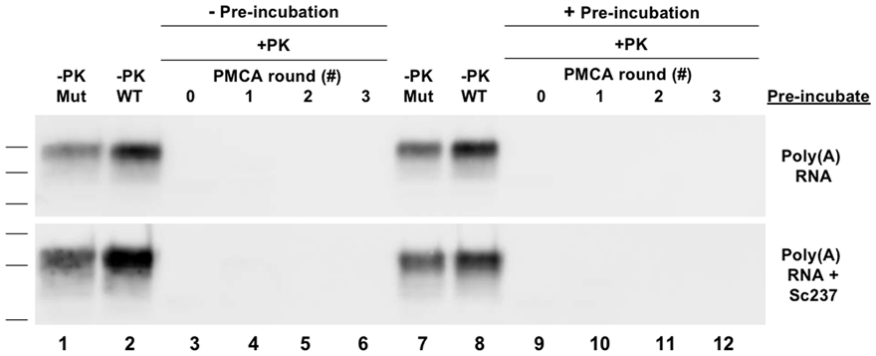
Inhibition of hamster PrP^C^ conversion following hamster PrP^C^ substrate pre-incubation with poly(A) RNA and/or Sc237. Western blots showing Sc237-seeded sPMCA propagation reactions containing wild type, Q172R HaPrP substrates, and synthetic poly(A) RNA. Reactions containing both CHO-expressed wild type HaPrP^C^ and the Q172R mutant HaPrP substrates at ∼1∶2 (Mut∶WT) ratio (*lanes 3–6* and *9–12*) were subjected to three rounds of serial propagation. Wild type HaPrP^C^ substrate was either pre-incubated (*+Pre-incubation*) or not (*−Pre-incubation*) with poly(A) RNA alone (*top blot*) or in combination with the Sc237 scrapie seed (*bottom blot*), as indicated, prior to addition of the Q172R HaPrP substrate and other components to the reaction. In all blots, samples containing recombinant wild type or mutant HaPrP substrate not subjected to proteinase K digestion are shown as a reference for comparison of electrophoretic mobility (*−PK WT or Mut*, respectively). All other samples were subjected to limited proteolysis with 50 µg/ml proteinase K for 1 hr at 37°C (*+PK*).

As there are several possible explanations for how Q172R HaPrP exerts its dominant negative properties, we sought to examine more closely the mechanism by which Q172R HaPrP-mediated inhibition occurs. One possible mechanism is that compared to wild type HaPrP^C^, Q172R HaPrP has increased affinity for binding poly(A) RNA, a necessary cofactor for conversion in this *in vitro* assay. To test whether Q172R HaPrP might interfere with the interaction between poly(A) RNA and wild type HaPrP^C^, we conducted a serial propagation reaction in which CHO-expressed wild type HaPrP^C^ substrate was allowed to pre-incubate with poly(A) RNA prior to addition of the Q172R HaPrP substrate to the reaction (Figure 5, *top blot*). We previously determined that our wild type HaPrP^C^ rapidly (<15 min) binds to immobilized poly(A) RNA (data not shown) and therefore, under the tested pre-incubation conditions, the wild type HaPrP^C^ substrate should have sufficient time to physically interact with poly(A) RNA before encountering Q172R HaPrP substrate. As previously demonstrated, when wild type and Q172R HaPrP substrates are combined simultaneously in the reaction, conversion of wild type HaPrP^C^ substrate is inhibited (Figure 4). When wild type HaPrP substrate was allowed to incubate with poly(A) RNA before the addition of Q172R HaPrP substrate and Sc237 seed, conversion of wild type HaPrP^C^ substrate was still inhibited (Figure 5, *+Pre-incubation*, *lanes 10–12*). Assuming that the wild type HaPrP^C^ substrate interacted with poly(A) RNA during the pre-incubation, this finding indicates that Q172R HaPrP substrate does not block conversion of wild type HaPrP^C^ substrate by sequestering the required cofactor, poly(A) RNA.

Another potential mechanism of Q172R HaPrP dominant negative inhibition is that, during the first round of sPMCA, this mutant binds to and sequesters the Sc237 scrapie template seed, making it inaccessible to wild type HaPrP^C^. Alternatively, Q172R HaPrP might bind directly to wild type HaPrP^C^ to physically block it from interacting with the scrapie seed. In either scenario, if the scrapie seed is physically inaccessible to wild type HaPrP^C^, then lack of interaction with this template could explain the inhibition of conversion. To test whether Q172R HaPrP prohibits wild type HaPrP^C^ from interacting with the scrapie seed, we performed a serial propagation reaction in which CHO-expressed wild type HaPrP^C^ substrate was pre-incubated with both poly(A) RNA and the Sc237 template seed (Figure 5, *bottom blot*). Again, without pre-incubation wild type HaPrP^C^ substrate failed to undergo conversion when added to the reaction simultaneously with the Q172R HaPrP substrate (Figure 5, *bottom blot, lanes 4–6*). Likewise, pre-incubating wild type HaPrP^C^ substrate with both Sc237 seed and poly(A) RNA before addition of Q172R HaPrP substrate to the reaction failed to rescue conversion (Figure 5, *bottom blot*, *lanes 10–12*). This result suggests that Q172R HaPrP inhibits prion formation by a mechanism other than preventing the interaction of wild type HaPrP^C^ with the Sc237 scrapie seed. However, a limitation of this experiment is that the initial binding of PrP^C^ to PrP^Sc^ might be weak, accompanied by a high dissociation rate. In this scenario, pre-incubation of mutant PrP molecules with the Sc237 scrapie seed might not prevent the template PrP^Sc^ molecules from subsequently interacting with wild type PrP^C^ molecules.

### Cross-seeding of wild type and mutant PrP molecules

**Figure 6:**
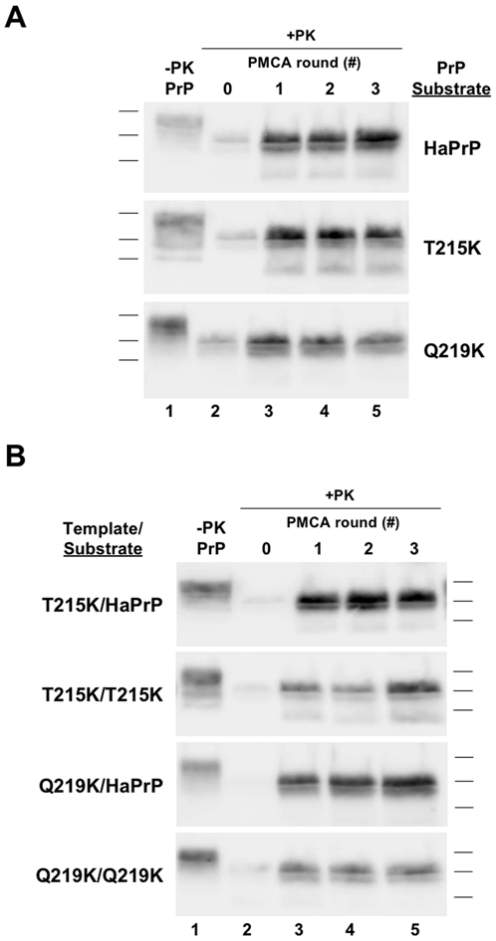
Cross-seeding experiments. *(A)* Reactions containing either wild type or mutant HaPrP substrate were originally seeded with CHO-derived HaPrPres and propagated for three rounds of sPMCA. *(B)* Reactions containing either wild type HaPrP^C^, T215K, or Q219K HaPrP substrates (*Substrate*) were originally seeded with CHO-derived T215K or Q219K HaPrPres template (*Template*) and subjected to three rounds of serial propagation. All reactions were supplemented with synthetic poly(A) RNA. In all blots, a sample containing wild type or mutant HaPrP substrate not subjected to proteinase K digestion is shown in the lane(s) preceding the corresponding PK-digested samples as a reference for comparison of electrophoretic mobility (*−PK PrP or Mut*). All other samples were subjected to limited proteolysis with 50 µg/ml proteinase K for 1 hr at 37°C (*+PK*).

Our finding that dominant negative inhibition of prion formation does not appear to involve the scrapie template or an external cofactor supports a model of inhibition mediated by direct interaction between PrP molecules. One possibility is that dominant negative PrP mutants cannot act as templates for conversion of wild type PrP. Therefore, if a mutant PrP molecule is integrated into a growing oligomer of converted wild type PrP, it could block further wild type PrP conversion because it lacks templating activity. In order to test whether dominant negative PrP mutants can act as a template for conversion of wild type PrP we conducted sPMCA propagation reactions containing wild type and mutant HaPrP substrates which were seeded with CHO-derived wild type or mutant HaPrPres (Figure 6). As expected, CHO-derived wild type HaPrPres was able to act as a template for the conversion of wild type, T215K, and Q219K HaPrP substrates (Figure 6A, *lanes 3–5*). Interestingly, T215K and Q219K HaPrPres were also able to act as a template for the conversion of wild type HaPrP^C^ substrate (Figure 6B, *top and lower middle blot*, *lanes 3–5*). As dominant negative HaPrP mutants retain the ability to seed the conversion of wild type HaPrP^C^ substrate, they must inhibit prion formation by a mechanism other than simply lacking templating activity.

## Discussion

### Infectious prions produced from CHO-expressed PrP substrate

In this study we report the development of a novel system for rapidly studying the effect of PrP mutations on prion propagation *in vitro*. Specifically, this system allows for characterizing the biochemical properties of mutant PrP while measuring the ability of mutant PrP to undergo conformational conversion to generate PrPres *in vitro*. This technique utilizes Flp-In CHO cells to generate cultured lines stably expressing PrP. Unlike bacterial expression systems, CHO cells process PrP in a more native manner that allows for the addition of a GPI anchor and N-linked carbohydrate groups. We found that PrP expressed in CHO cells displayed an aberrant glycosylation profile relative to native, brain-derived PrP^C^. However, despite this difference in processing, we showed that partially purified wild type HaPrP^C^ and MoPrP^C^ substrates expressed in CHO cells could be converted to a protease-resistant and self-propagating PrP conformation using the sPMCA assay. The PrPres formed during PMCA was as equally resistant to PK treatment as PrP^Sc^ formed in sPMCA using brain-derived PrP^C^ substrates [11],[12]. In addition, we found that CHO-expressed HaPrP^C^ substrate could be converted *in vitro* to HaPrP^Sc^, which was autocatalytic and infectious to wild type hamsters, with associated strain properties indistinguishable from Sc237-seeded, sPMCA-generated, brain-derived HaPrP^Sc^ molecules. The 140 day prion incubation time produced by Sc237-seeded, CHO-expressed, sPMCA-generated HaPrP^Sc^ molecules was significantly longer than that produced by native Sc237 prions, and slightly shorter than that produced by Sc237-seeded, brain-derived HaPrP^Sc^ molecules [11],[38]. Based on the previously measured relationship between titer and incubation time for brain-derived, sPMCA-generated HaPrP^Sc^ molecules [11] and quantitation of PrP concentration by serial dilutions on Western blots, we calculate that the specific infectivity of CHO-derived, sPMCA-generated HaPrP^Sc^ molecules is approximately equal to that of a brain-derived preparation of PrP27-30 molecules. It is not known why sPMCA-generated HaPrP^Sc^ molecules exhibit an altered relationship between titer and incubation time, but studies with nitrocellulose carriers suggest that sonication may disrupt the size distribution of infectious HaPrP^Sc^ molecules [40]. Thus, with these caveats taken into consideration, our findings show for the first time that PrP^C^ expressed in a cultured mammalian cell line can be used as a substrate for the generation of an infectious prion *in vitro*. This indicates that utilizing CHO-expressed PrP^C^ as an sPMCA substrate is a powerful approach for studying the molecular mechanism of prion formation *in vitro*.

Furthermore, combining PrP expressed in cell culture with the sPMCA technique provides several advantages over other methods of studying prion conversion. In cell-based models of prion propagation, cellular processes such as PrP trafficking and clearance of PrP^Sc^ can pose a challenge to study the conversion of PrP effectively. The system we describe allows for the study of PrP in the absence of any cellular process that might influence prion conversion. Many of the cell lines that can propagate prions, such as mouse N2a cells, express endogenous PrP^C^ [14],[48]. As such, PrP expressed in these lines must often be tagged in some manner in order to discriminate it from endogenous PrP^C^. This does not pose a problem in our system because CHO cells do not express detectable levels of endogenous PrP^C^ [41]. In addition to studying PrP mutations, prion strains and species differences can be easily investigated using this system, providing a versatile and efficient alternative to studying PrP in cell culture or transgenic mouse models of prion conversion. However, in implementing this system in our studies, we are aware that the relationship between PrP and prion infectivity is complex, and remains poorly understood. Some disease models have little or no detectable PrP^Sc^ despite high levels of prion infectivity [5],[6], and the measurement of protease-resistant PrP^Sc^ by Western blotting (without amplification) is not as sensitive as bioassay. In our system, some of these issues are at least partially mitigated by the use of 3-day sPMCA assays, which are highly sensitive [49], report upon PrP^Sc^ autocatalysis in addition to protease-resistance, and allow time for slowly propagating reactions between heterologous PrP molecules to adapt conformation. Nonetheless, like all biochemical assays of prion formation, sPMCA experiments must be used and interpreted cautiously.

### Species-dependent effects of putative protein X binding site mutation

Previous studies in transgenic mouse and cell-culture models of prion conversion led to the identification of four residues within MoPrP which, when mutated singly to basic residues, prevented prion formation [13],[14]. Utilizing PrP expressed in CHO cells, we found that Q171R, V214K, and Q218K MoPrP substrates could not be converted into MoPrPres in sPMCA reactions supplemented with Prnp^0/0^ brain homogenate, confirming that residues Q171, V214, and Q218 are also required for the formation of RML mouse prions *in vitro*. In contrast, two hamster PrP mutants (T215K and Q219K), whose corresponding MoPrP homologues do not support prion formation either in RML-seeded sPMCA reactions or in the ScN2a cell-culture model, could be readily converted into self-propagating PrPres in sPMCA reactions driven by Sc237 hamster scrapie. These opposing results could be due to either differences in the species of the PrP substrate or the scrapie strain used to seed the propagation reactions. In either case, the ability of hamster T215K and Q219K PrP to convert into autocatalytic PrPres is inconsistent with the hypothesis that these residues are required to help form a discontinuous binding site for an accessory catalyst shared among rodent species, such as protein X. Moreover, the differences in behavior between mouse and hamster PrP mutants also suggest that the mechanism of prion formation may not be identical between these two species. Indeed, previous studies have shown that whereas formation of mouse prions *in vitro* requires unglycosylated MoPrP^C^ molecules in the substrate, unglycosylated HaPrP^C^ molecules inhibit the formation of hamster prions in a dose-dependent fashion [39], and that recombinant hamster and mouse PrP molecules follow different folding pathways towards amyloid fibril formation [50]. Interestingly, Hizume *et al.* recently reported that transgenic mice expressing human PrP E219K were susceptible to human prions [23]. Therefore, this mutation does not abolish the ability of PrP molecules from at least two different animal species to act as substrates for prion formation.

Our studies also showed that residue Q171 in mouse PrP and the homologous residue Q172 in hamster PrP was required for prion formation in both species, raising the possibility that this residue may play a fundamental role in the process of prion formation in both species.

### PrP mutants retain dominant inhibitory activity *in vitro*

**Figure 7:**
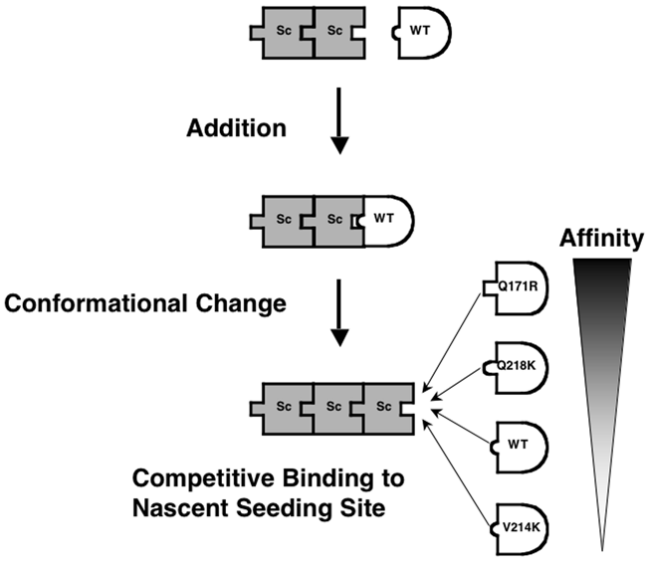
Model of *trans*-dominant inhibition of prion formation. This diagram illustrates the proposed model of competition between various PrP substrate molecules for binding to a nascent seeding site on newly formed PrP^Sc^ molecules. Legend: Sc = PrP^Sc^; WT = wild type PrP^C^; mutant PrP molecules labeled according to residue numbering for mouse PrP. The relative affinities of various PrP molecules for the nascent seeding site on the growing PrP^Sc^ polymer are indicated schematically by the length and curvature of the cartoon protrusion, which is meant to represent the NSS binding domain, and which likely includes the residue 170–174 loop domain.

Collectively, the data presented in this manuscript refute the hypothesis that protein X or any other accessory cofactor is required for dominant negative inhibition of prion formation. Moreover, the ability of the Q172R mutant to inhibit hamster prion formation in a purified system even when WT HaPrP substrate was pre-incubated with both poly(A) RNA and scrapie template suggests that mutant PrP inhibits the conversion of WT PrP by blocking a nascent seeding site (NSS) on newly formed, PMCA-generated PrP^Sc^ molecules (Figure 7). Our data also show complete dissociation between conversion ability and *trans*-dominant inhibitory activity. Most notably, we showed that Q219K HaPrP is able both to convert into PrPres itself and to inhibit the conversion of WT PrP in *trans*. Thus, we can conclude that the mechanism of *trans*-dominant inhibition does not require a “negative” mutation, i.e. a PrP molecule that is unable to convert into PrPres itself.

The simplest model that can account for these observations simultaneously is that the tested PrP mutants directly compete for binding to the PrP^Sc^ NSS, regardless of their ability to convert to PrPres (Figure 7). Because Q172R is able to act as a dominant negative inhibitor at sub-stoichiometric levels, we reason that this mutant has the highest affinity for the PrP^Sc^ NSS amongst the mutants tested. In contrast, because T215K does not appear to inhibit conversion of WT PrP in *trans*, we reason that this mutant has the lowest affinity for the PrP^Sc^ NSS amongst the mutants tested. Because Q219K HaPrP only blocks conversion of WT HaPrP when the concentration of mutant protein is in molar excess, we reason that Q219K HaPrP (and by extension, the homologous Q218K MoPrP mutant) has lower affinity for the PrP^Sc^ NSS than the Q172R. We can also deduce that Q219K HaPrP has a higher affinity for the PrP^Sc^ NSS than WT HaPrP because WT PrP does not dominantly inhibit the conversion of Q219K PrP, even when the concentration of WT PrP exceeds that of Q219K. Taken together, we propose the following rank order of affinity for the PrP^Sc^ NSS (numbering based on mouse PrP): Q171R>Q218K>WT≥V214K (Figure 7).

It is interesting to speculate about the possible mechanism responsible for the high affinity of the Q171R mutant. Residue Q171 resides within a short loop that connects β2 strand and α2 helix (residues 170–174), which has been implicated in the transmission barrier between animal species and in the *de novo* formation of chimeric cervid/mouse prions [51],[52]. Moreover, the high affinity of Q171R suggests that this loop may also be the major PrP^C^ epitope recognized by the PrP^Sc^ NSS. Q171R may bind with higher affinity than WT PrP^C^ because the PrP^Sc^ NSS may contain an anionic contact point, either due to exposure of a negatively charged PrP residue, or of a physically embedded anionic cofactor [12]. Both the high affinity of the Q171R mutant for the PrP^Sc^ NSS and the ability of the Q171R mutation to prevent conversion of both hamster and mouse PrP molecules (as described in the preceding section) lend support to the hypothesis proposed by Sigurdsson *et al.* that the loop region which contains this residue plays a vital role in prion formation [51].

Using an *in vitro* fibril formation technique, Lee *et al.* showed that mixtures of bacterially-expressed, recombinant WT and Q218K MoPrP molecules displayed slower polymerization kinetics than samples containing either WT or Q218K MoPrP alone [30]. It is likely that heteropolymer destabilization also contributes to the ability of *trans*-dominant PrP mutants to inhibit the sPMCA-facilitated propagation of mammalian prions, under conditions that permit both mutant and WT PrP molecules to bind to and incorporate into the growing PrP^Sc^ polymer. However, the process of heteropolymer destabilization alone cannot fully explain the results of our studies, particularly the asymmetrical interaction between WT and Q219K HaPrP (i.e., Q219K completely inhibits conversion of WT HaPrP when present in molar excess, but not *vice versa*, indicating that WT PrP is excluded from co-polymerizing with Q219K PrP when the mutant is present in excess (Figure 4)). Hence, we propose that *trans*-dominant inhibition potentially occurs at two sequential stages: (1) initially, mutant and WT PrP molecules compete to bind at the PrP^Sc^ NSS, and then (2) if both mutant and WT PrP molecules are able to bind to and incorporate into a growing PrP^Sc^ polymer, the heteropolymer may become kinetically destabilized.

Using scrapie-infected tissue culture models, other investigators have previously demonstrated dose-dependent, dominant negative inhibition by heterologous PrP^C^ molecules [31],[32]. Additional studies showed that the presence of non-converting, heterologous PrP^C^ molecules interfered with the ability of PrP^C^ molecules homologous to the PrP^Sc^ template to acquire protease-resistance in a cell-free assay using purified components [33]. Interestingly, radioactive heterologous and homologous PrP^C^ molecules bound to the PrP^Sc^ template equally well, suggesting two alternative models for heterologous interference [33],[53]: (1) in the single site model, inhibition is proposed to occur primarily by the inhibition of conversion (rather than binding) of homologous PrP^C^ substrate to PrP^Sc^ by heterologous, non-converting PrP^C^ molecules; (2) in the two-site model, it is proposed that heterologous PrP^C^ molecules competitively inhibit binding of homologous PrP^C^ substrate to a conversion-inducing site on the PrP^Sc^ template, while both species of PrP^C^ molecules are able to bind non-competitively to a second binding site on PrP^Sc^, which does not influence conversion. It is reasonable to hypothesize that the same molecular mechanism underlies interference between heterologous PrP^C^ molecules and the *trans*-dominant inhibitory activity of the “protein X binding site” mutants. If that hypothesis is correct, then it is interesting to note that several results from our study appear to be incompatible with the single site model; specifically we identified: (1) convertible PrP mutants that can inhibit conversion in trans; (2) non-convertible PrP mutants that do not inhibit in *trans*; (3) a dominant negative mutant that inhibits at sub-stoichiometric levels; and (4) asymmetric inhibition between two convertible PrP^C^ molecules. These results would be compatible with the two-site model, in which the NSS would represent the conversion-inducing site. However, further studies are required to confirm the hypothesis that the same mechanism mediates the inhibitory activities of heterologous PrP^C^ molecules and “protein X binding site” mutants.

In summary, our results show that protein X is not required to mediate dominant inhibition of prion propagation *in vitro*, and suggest instead that PrP molecules compete for binding to the NSS of newly formed PrP^Sc^ molecules. A critical part of the binding surface appears to be located within the short loop (between β2 strand and α2 helix) of PrP^C^ recently shown to modulate spontaneous prion formation [51]. However, the results of biochemical assays cannot be equated to the genetic control of prion transmission *in vivo*, and further studies in living animals will be required to confirm the physiological basis of dominant negative inhibition.

## Supporting Information

Text S1 Supplemental Methods
(0.10 MB PDF)

Figure S1 Stable expression of PrP molecules in CHO cells. Western blot showing samples containing either brain-derived MoPrP^C^ (*lane 1*) and HaPrP^C^ (*lane 6*) or CHO-expressed wild type, Q171R, V214K, and Q218K MoPrP (*lanes 2–5*, respectively) and wild type, Q172R, T215K, and Q219K HaPrP (*lanes 7–10*, respectively). The PrP in all samples was partially purified on a cobalt-IMAC column before immunoblot detection.
(0.29 MB PDF)

Figure S2 Regional neuropathology of hamsters inoculated with *in vitro*-generated PrP^Sc^ molecules. Vacuolation profile scores (A) and PrP 3F4 immunohistochemistry profiles (B) of animals inoculated with samples containing PrP^Sc^ molecules generated *in vitro* from Sc237-seeded 15-cycle sPMCA reactions containing PrP^C^ (open squares) prepared from stably transfected CHO cells or (filled circles) isolated from hamster brain. For each, the mean values (*n* = 6–15 animals/group) are shown ±SEM. Brain regions: FC, frontal cortex; PC, parietal cortex; H, hippocampus; C, cerebellum; M, medulla.
(0.31 MB PDF)

Figure S3 sPMCA propagation reactions seeded with the 139H scrapie strain. Western blots showing sPMCA propagation reactions containing wild type, Q172R, T215K, or Q219K mutant HaPrP substrates. Reactions containing either wild type or mutant HaPrP substrate alone were originally seeded with 139H scrapie brain homogenate and propagated for three rounds of sPMCA. All reactions were supplemented with synthetic poly(A) RNA. In all blots, a sample containing wild type or mutant HaPrP substrate not subjected to proteinase K digestion is shown in the lanes preceding the corresponding PK-digested samples as a reference for comparison of electrophoretic mobility (*PrP, Mut −PK*). All other samples were subjected to limited proteolysis with 50 µg/ml proteinase K for 1 hr at 37°C (+*PK*).
(0.27 MB PDF)

Figure S4 Effect of increasing propagation time length on mutant PrP conversion and inhibition. Reactions containing either wild type (*WT only, lanes 2–5*), an equimolar mixture of wild type and Q172R HaPrP (*WT* +*Mutant, lanes 8–11*), or Q172 HaPrP (*Mutant only, lanes 13–16*) substrate alone were originally seeded with Sc237 scrapie brain homogenate and propagated for three rounds of sPMCA. All reactions were supplemented with synthetic poly(A) RNA and originally seeded with Sc237 brain homogenate. The time length of an individual propagation round was 1 day (top blot), 2 days (middle blot), or 4 days (bottom blot). In all blots, a sample containing wild type or mutant HaPrP substrate not subjected to proteinase K digestion is shown in the lane(s) preceding the corresponding PK-digested samples as a reference for comparison of electrophoretic mobility (*−PK WT* or *Mut*). All other samples were subjected to limited proteolysis with 50 µg/ml proteinase K for 1 hr at 37°C (+*PK*).
(0.38 MB PDF)

Figure S5 Cell surface localization of HaPrP molecules expressed in CHO cells. CHO cell lines stably expressing wild type and mutant HaPrP were treated with (*lanes 3* and *4*) or without (*lanes 1* and *2*) PI-PLC in order to assess anchorage to the outer leaflet of the plasma membrane via the GPI anchor. Following treatment, proteins in the PIPLC incubation media (*Media, lanes 1* and *3*) were precipitated, and the cells (*Lysate, lanes 2* and *4*) were harvested in lysis buffer. PrP was detected in the precipitated protein and cell lysate samples by Western blotting.
(0.32 MB PDF)

Figure S6 Detergent solubility of HaPrP molecules expressed in CHO cells. CHO cell lines stably expressing wild type and mutant HaPrP were harvested in lysis buffer containing 0.5% Triton-X 100 and 0.5% DOC. A portion of each cell lysate was removed (*TOT, lane 1*), the remainder of each lysate was centrifuged at 100,000×*g*, and the supernatant and pellet fractions were isolated. Equivalent amounts of the Total lysates, supernatant (*SUP, lane 2*) and pellet (*PEL, lane 3*) fractions were resuspended in SDS-PAGE loading buffer and rPrP was detected in each sample by Western blotting.
(0.22 MB PDF)
