# Supplementary material for: Trans-Dominant Inhibition of Prion Propagation In Vitro Is Not Mediated by an Accessory Cofactor
Source: PLoS Pathog. 2009 Jul 31;5(7):e1000535. doi: 10.1371/journal.ppat.1000535 (PMC2713408; doi:10.1371/journal.ppat.1000535)
Supplement: Text S1 — Supplemental Methods (0.10 MB PDF) [file ppat.1000535.s001.pdf]

Supplemental Methods

Reagents

Synthetic poly(A) catalog # P9403 was purchased from Sigma-Aldrich (St. Louis, MO). Stock solutions of synthetic polynucleotides were prepared in 1x TE pH 8.0, and OD_{260} readings prior to use confirmed concentrations. Deoxycholic acid (DOC), sodium salt catalog # D6750, MOPS catalog # M5789, MES catalog # M5287, cobalt chloride catalog # C-2644, and imidazole catalog # I-5513 were purchased from Sigma. RNase-free water and TE pH 8.0 were purchased from Ambion (Austin, TX). Other chemical reagents were purchased at the highest grade available from Fisher Scientific (Pittsburgh, PA). Ham's F12 media, Dulbecco's phosphate buffered saline (DPBS) and phosphate buffered saline (PBS) without calcium or magnesium was purchased from Mediatech, Inc. (Herndon, VA). Glycko® phosphatidylinositol phospholipase C (PI-PLC) catalog # GKGPI-02A was purchased from Prozyme (San Leandro, CA). The anti-PrP mAb, D13 and the anti-PrP mAb 27/33 were provided by Dr. Anthony Williamson (La Jolla, CA) and Dr. Peter Morganelli (VA Hospital, White River Junction, VT), respectively. Flp-In™ Chinese hamster ovary (CHO) cells catalog # R758-07, the Flp-In™ system catalog # K6010-01, including the expression vector pcDNA5/FRT and Flp-recombinase encoding plasmid pOG44, Lipofectamine™ 2000 catalog # 11668-019, OptiMEM® catalog # 11058, hygromycin B catalog # 10687-010, fetal bovine serum catalog # 16000-036, and T4 DNA ligase catalog # 15224 were obtained from Invitrogen (Carlsbad, CA). All restriction endonucleases were obtained from New England Biolabs. $PrP^{0/0}$ mice were kindly provided by David Harris with permission from Charles Weissmann [1]. Hamster scrapie strains Sc237, 139H and the mouse scrapie strain RML, were kindly provided by Dr. Stanley Prusiner (San Francisco, CA).

PrP Expression Plasmid Construction

The cloning plasmids p805/HaPrP and pCombo3/MoPrP (from Mike Scott, Dublin, Ireland) encoding the full-length coding sequence of wild type hamster and mouse PrP, were used as templates for generating mutant PrP constructs. The pCombo3/MoPrP plasmid encodes wild type MoPrP with methionine at position 128 and p805/HaPrP encodes wild type HaPrP with methionine at position 129. Mouse and hamster PrP mutants were generated using the Gene Tailor Site-Directed Mutagenesis System (Invitrogen). All wild type and mutant PrP coding sequences were excised from the

cloning plasmids by digestion with XhoI and BglII and then purified using a Qiagen gel extraction kit. Gel-purified fragments were inserted into the multiple cloning site of the pcDNA5/FRT expression vector, which was pre-digested with XhoI and BamHI to create matching ends. Plasmid DNA of each pcDNA5/FRT/PrP expression construct was prepared by transformation into DH5α T1 Max Efficiency cells (Invitrogen) followed by DNA isolation using a Qiagen endotoxin-free plasmid DNA purification kit.

***In vivo* infectivity assay**

To prepare the inoculum, a sPMCA reaction containing cobalt affinity column purified CHO-expressed $HaPrP^{C}$ substrate and synthetic poly(A) RNA was seeded with a 1:100 dilution of 10% crude Sc237 scrapie-brain homogenate and propagated for 15 rounds of sPMCA. As a control, a preparation of untransfected CHO cells purified on a cobalt affinity column was used as a sPMCA substrate instead of $HaPrP^{C}$; this substrate was also seeded with Sc237 and propagated for 15 rounds of sPMCA. The round 15 sPMCA reactions were subjected to ultracentrifugation at 100,000 x *g* and the pellets were resuspended in sterile PBS. Wild type Golden Syrian Hamsters were intracerebrally inoculated with 50 μl inoculum. Neuropathological analysis included hemotoxylin and eosin staining and immunohistochemical detection of PrP deposits with 3F4 mAb as previously described [2].

PrP^{Sc} detection assay

All protease digested (+PK) samples were incubated with either 50 μg/ml Proteinase K (Roche) for 1 h at 37°C or 25 μg/ml Proteinase K for 30 min at 37°C for hamster and mouse PrP samples, respectively. An equal volume of 2x SDS sample buffer was then added and samples were boiled for 10 min at 95°C. SDS-PAGE was performed on 1.5-mm 12% polyacrylamide gels with an acrylamide:bisacrylamide ratio of 29:1 (Bio-Rad). Following electrophoresis, the proteins were transferred to a methanol-charged, buffer equilibrated polyvinylide difluoride (PVDF) membrane (Millipore) using a Trans-blot SD Semi-Dry Transfer Cell (Bio-Rad) set at 2 mA/cm^2 for 30 min.

To visualize PrP signals Western blot membranes were rinsed with TBST (10 mM Tris pH 7.2, 150 mM NaCl, 0.1% Tween-20) and blocked for 1 h in Hood (Chelsea, MA) skim milk buffered with TBST. The blocked membrane was incubated overnight at 4°C with either 6D11 mAb (Signet), D13 mAb, or 27/33 mAb diluted 1:25,000, 1:2500 or 1:25,000

respectively, in TBST. Following this incubation, the membrane was washed 3 x 10 min in TBST, and incubated for 1 h at 4°C with either horseradish peroxidase-labeled anti-mouse or anti-human IgG secondary antibody conjugate (GE Healthcare and Pierce) diluted 1:5000 and 1:20,000, respectively in TBST. The membrane was washed again 4 x 10 min with TBST. The blot was developed using West Femto (Pierce) chemiluminescence substrate and captured digitally using a Fuji (Fujifilm) LAS-3000 chemiluminescence system. Digital images were captured using Image Reader version 2.0 (Fujifilm, Tokyo, Japan). Relative molecular masses were based on migration of pre-stained standards from either Fermentas (Hanover, MD) or Bio-Rad (Hercules, CA). On all blots, molecular masses of approximately 43, 34, and 26 kDa are indicated by three dashes.

CHO-Expressed PrP solubility assay

CHO cell lines stably expressing PrP were grown to confluence in 60mm dishes (Corning). Each dish of cells was washed once with PBS and harvested using 1 ml of cell lysis buffer (150mM NaCl, 50mM Tris HCL pH 7.5, 0.5% Triton-X 100, 0.5% DOC, plus Complete™ protease inhibitors). Cell lysates were pre-cleared by centrifugation at 16,000 x *g* for 5 min at 4 °C. To prepare a sample representing the total cell lysate, 100 μl of each pre-cleared cell lysate was removed, combined with 100 μl 2X SDS loading buffer and boiled. The remainder of each pre-cleared lysate was subjected to ultracentrifugation at 100,000 x *g* for 1 hr at 4 °C. Sample supernatants containing detergent-soluble PrP were combined with an equal volume of 2X SDS loading buffer and boiled for 10 min at 95 °C. Pellet fractions containing detergent-insoluble PrP were resuspended in a volume of cell lysis buffer equal to the removed supernatant fraction and then combined with an equal volume of 2X SDS loading buffer and boiled. All samples were then analyzed for the presence of PrP by Western blot using the mAbs, 6D11 or D13.

CHO-Expressed PrP PI-PLC release assay

CHO cell lines stably expressing PrP were grown to confluence in 6-well dishes (Corning). Cells were washed with DPBS and 1 ml of OptiMEM® containing either no PI-PLC or 1U/ml PI-PLC was added to the cells, depending on the condition. Cells were

returned to a 37 °C incubator for 1 hr, after which the OptiMEM® media was removed from each well and the proteins present in the media were precipitated using the method of Wessel and Flugge [3]. Precipitated protein pellets were solubilized in 100 μl 2X SDS loading buffer and boiled for 10 min at 95 °C. Cells in the dish were rinsed with PBS and harvested using 0.5 ml of cell lysis buffer (150mM NaCl, 50mM Tris HCL pH 7.5, 0.5% Triton-X 100, 0.5% DOC, plus Complete™ protease inhibitors) per well. 100 μl of each lysate was combined with an equal volume of 2X SDS loading buffer and boiled. All samples were analyzed for the presence of PrP by Western blot using the mAb, D13.

References

1. Bueler H, Fischer M, Lang Y, Bluethmann H, Lipp HP, et al. (1992) Normal development and behaviour of mice lacking the neuronal cell- surface PrP protein. Nature 356: 577-582.
2. Deleault NR, Harris BT, Rees JR, Supattapone S (2007) Formation of native prions from minimal componenets in vitro. Proc Natl Acad Sci U S A 104: 9741-9746.
3. Wessel D, Flugge UI (1984) A method for the quantitative recovery of protein in dilute solution in the presence of detergents and lipids. Anal Biochem 138: 141-143.
