# Supplementary material for: Trans-Dominant Inhibition of Prion Propagation In Vitro Is Not Mediated by an Accessory Cofactor
Source: PLoS Pathog. 2009 Jul 31;5(7):e1000535. doi: 10.1371/journal.ppat.1000535 (PMC2713408; doi:10.1371/journal.ppat.1000535)
Supplement: Figure S1 — Stable expression of PrP molecules in CHO cells. Western blot showing samples containing either brain-derived MoPrPC (lane 1) and HaPrPC (lane 6) or CHO-expressed wild type, Q171R, V214K, and Q218K MoPrP (lanes 2–5, respectively) and wild type, Q172R, T215K, and Q219K HaPrP (lanes 7–10, respectively). The PrP in all samples was partially purified on a cobalt-IMAC column before immunoblot detection. (0.29 MB PDF) [file ppat.1000535.s002.pdf]

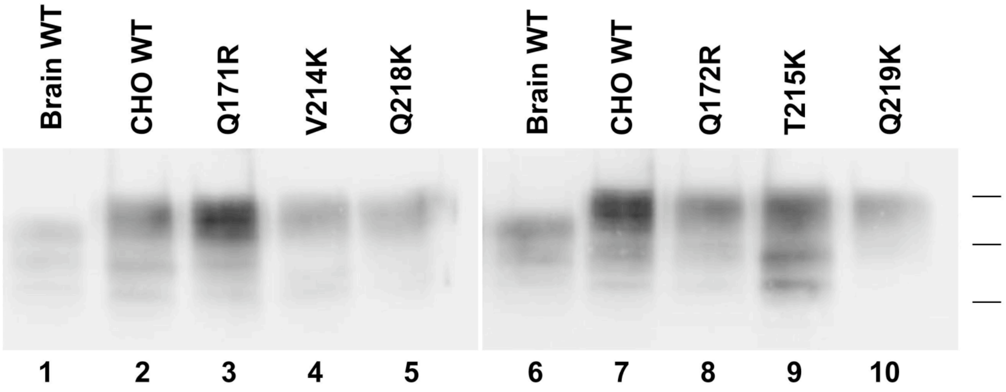

Figure S1.

Stable expression of PrP molecules in CHO cells.

Western blot showing samples containing either brain-derived $MoPrP^C$ (*lane 1*) and $HaPrP^C$ (*lane 6*) or CHO-expressed wild type, Q171R, V214K, and Q218K MoPrP (*lanes 2-5*, respectively) and wild type, Q172R, T215K, and Q219K HaPrP (*lanes 7-10*, respectively). The PrP in all samples was partially purified on a cobalt-IMAC column before immunoblot detection.
