# Supplementary material for: Trans-Dominant Inhibition of Prion Propagation In Vitro Is Not Mediated by an Accessory Cofactor
Source: PLoS Pathog. 2009 Jul 31;5(7):e1000535. doi: 10.1371/journal.ppat.1000535 (PMC2713408; doi:10.1371/journal.ppat.1000535)
Supplement: Figure S2 — Regional neuropathology of hamsters inoculated with in vitro-generated PrPSc molecules. Vacuolation profile scores (A) and PrP 3F4 immunohistochemistry profiles (B) of animals inoculated with samples containing PrPSc molecules generated in vitro from Sc237-seeded 15-cycle sPMCA reactions containing PrPC (open squares) prepared from stably transfected CHO cells or (filled circles) isolated from hamster brain. For each, the mean values (n = 6–15 animals/group) are shown ±SEM. Brain regions: FC, frontal cortex; PC, parietal cortex; H, hippocampus; C, cerebellum; M, medulla. (0.31 MB PDF) [file ppat.1000535.s003.pdf]

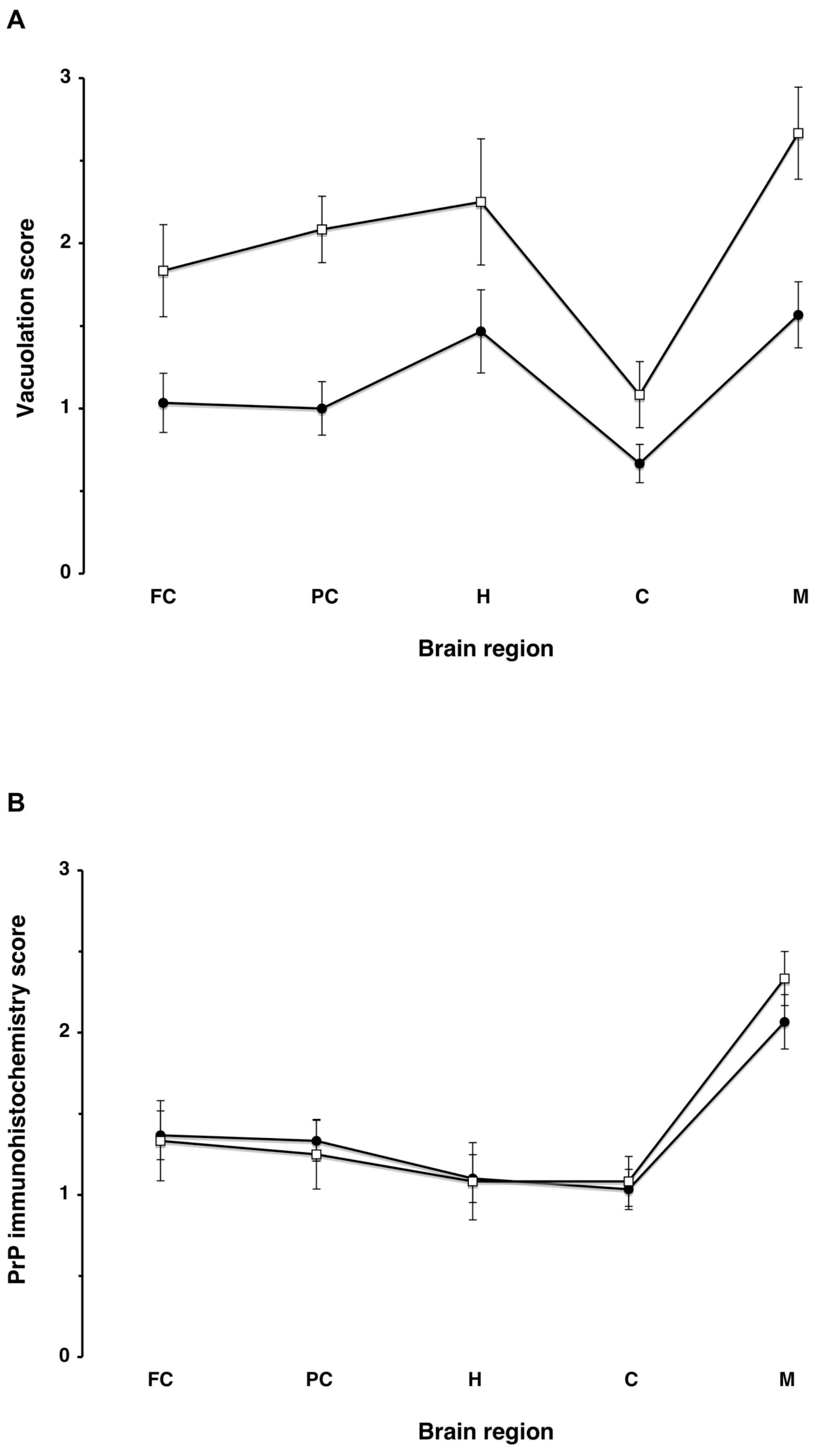
A
Vacuolation score
3
2
1
0
FC
PC
H
C
M
Brain region
B
PrP immunohistochemistry score
3
2
1
0
FC
PC
H
C
M
Brain region

Figure S2.

Regional neuropathology of hamsters inoculated with *in vitro*-generated PrP^{Sc} molecules.

Vacuolation profile scores (*A*) and PrP 3F4 immunohistochemistry profiles (*B*) of animals inoculated with samples containing PrP^{Sc} molecules generated *in vitro* from Sc237-seeded 15-cycle sPMCA reactions containing PrP^{C} (open squares) prepared from stably transfected CHO cells or (filled circles) isolated from hamster brain. For each, the mean values (n = 6-15 animals/group) are shown ±SEM. Brain regions: FC, frontal cortex; PC, parietal cortex; H, hippocampus; C, cerebellum; M, medulla.
