# Supplementary material for: Trans-Dominant Inhibition of Prion Propagation In Vitro Is Not Mediated by an Accessory Cofactor
Source: PLoS Pathog. 2009 Jul 31;5(7):e1000535. doi: 10.1371/journal.ppat.1000535 (PMC2713408; doi:10.1371/journal.ppat.1000535)
Supplement: Figure S3 — sPMCA propagation reactions seeded with the 139H scrapie strain. Western blots showing sPMCA propagation reactions containing wild type, Q172R, T215K, or Q219K mutant HaPrP substrates. Reactions containing either wild type or mutant HaPrP substrate alone were originally seeded with 139H scrapie brain homogenate and propagated for three rounds of sPMCA. All reactions were supplemented with synthetic poly(A) RNA. In all blots, a sample containing wild type or mutant HaPrP substrate not subjected to proteinase K digestion is shown in the lanes preceding the corresponding PK-digested samples as a reference for comparison of electrophoretic mobility (PrP, Mut −PK). All other samples were subjected to limited proteolysis with 50 µg/ml proteinase K for 1 hr at 37°C (+PK). (0.27 MB PDF) [file ppat.1000535.s004.pdf]

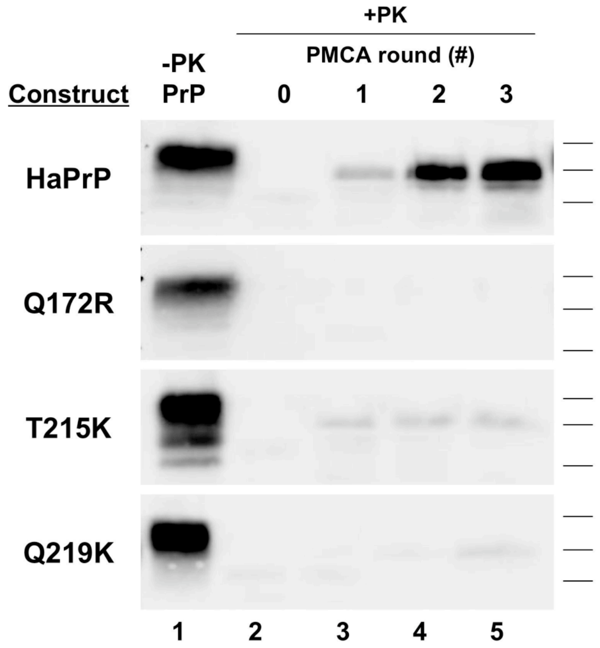

Figure S3.

sPMCA propagation reactions seeded with the 139H scrapie strain.

Western blots showing sPMCA propagation reactions containing wild type, Q172R, T215K, or Q219K mutant HaPrP substrates. Reactions containing either wild type or mutant HaPrP substrate alone were originally seeded with 139H scrapie brain homogenate and propagated for three rounds of sPMCA. All reactions were supplemented with synthetic poly(A) RNA. In all blots, a sample containing wild type or mutant HaPrP substrate not subjected to proteinase K digestion is shown in the lanes preceding the corresponding PK-digested samples as a reference for comparison of electrophoretic mobility (*PrP, Mut -PK*). All other samples were subjected to limited proteolysis with 50 μg/ml proteinase K for 1 hr at 37°C (+*PK*).
