# Supplementary material for: Trans-Dominant Inhibition of Prion Propagation In Vitro Is Not Mediated by an Accessory Cofactor
Source: PLoS Pathog. 2009 Jul 31;5(7):e1000535. doi: 10.1371/journal.ppat.1000535 (PMC2713408; doi:10.1371/journal.ppat.1000535)
Supplement: Figure S4 — Effect of increasing propagation time length on mutant PrP conversion and inhibition. Reactions containing either wild type (WT only, lanes 2–5), an equimolar mixture of wild type and Q172R HaPrP (WT +Mutant, lanes 8–11), or Q172 HaPrP (Mutant only, lanes 13–16) substrate alone were originally seeded with Sc237 scrapie brain homogenate and propagated for three rounds of sPMCA. All reactions were supplemented with synthetic poly(A) RNA and originally seeded with Sc237 brain homogenate. The time length of an individual propagation round was 1 day (top blot), 2 days (middle blot), or 4 days (bottom blot). In all blots, a sample containing wild type or mutant HaPrP substrate not subjected to proteinase K digestion is shown in the lane(s) preceding the corresponding PK-digested samples as a reference for comparison of electrophoretic mobility (−PK WT or Mut). All other samples were subjected to limited proteolysis with 50 µg/ml proteinase K for 1 hr at 37°C (+PK). (0.38 MB PDF) [file ppat.1000535.s005.pdf]

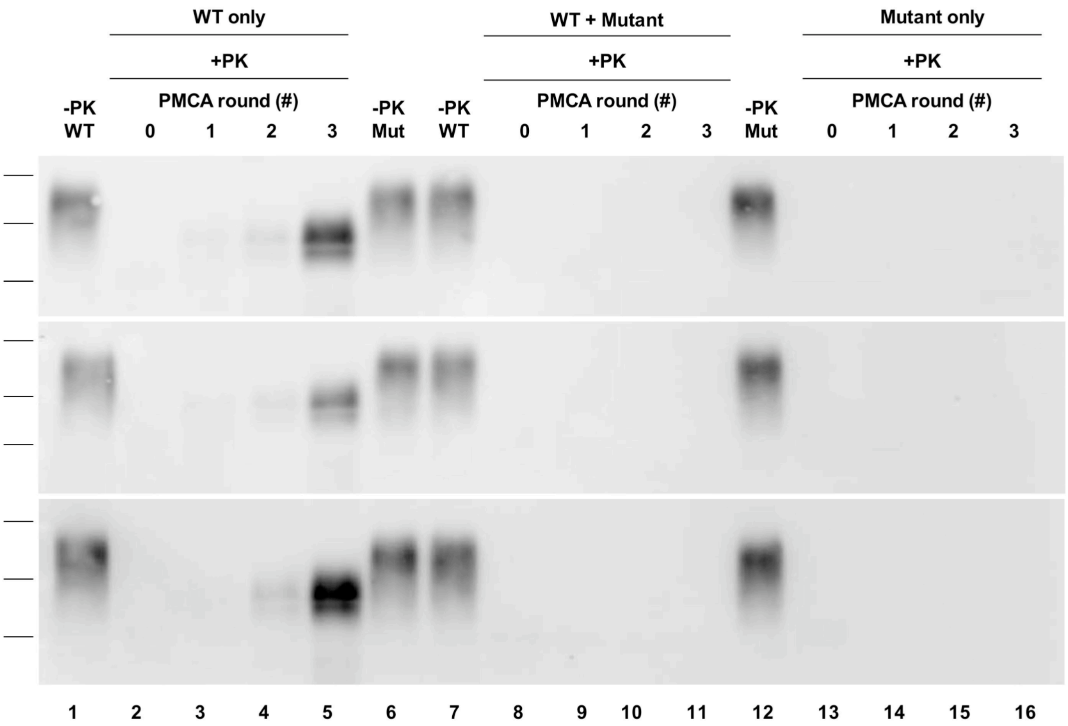

Figure S4.

Effect of increasing propagation time length on mutant PrP conversion and inhibition.

Reactions containing either wild type (*WT only, lanes 2-5*), an equimolar mixture of wild type and Q172R HaPrP (*WT + Mutant, lanes 8-11*), or Q172 HaPrP (*Mutant only, lanes 13-16*) substrate alone were originally seeded with Sc237 scrapie brain homogenate and propagated for three rounds of sPMCA. All reactions were supplemented with synthetic poly(A) RNA and originally seeded with Sc237 brain homogenate. The time length of an individual propagation round was 1 day (top blot), 2 days (middle blot), or 4 days (bottom blot). In all blots, a sample containing wild type or mutant HaPrP substrate not subjected to proteinase K digestion is shown in the lane(s) preceding the corresponding PK-digested samples as a reference for comparison of electrophoretic mobility (*-PK WT or Mut*). All other samples were subjected to limited proteolysis with 50 μg/ml proteinase K for 1 hr at 37°C (*+PK*) .
