# Supplementary material for: Trans-Dominant Inhibition of Prion Propagation In Vitro Is Not Mediated by an Accessory Cofactor
Source: PLoS Pathog. 2009 Jul 31;5(7):e1000535. doi: 10.1371/journal.ppat.1000535 (PMC2713408; doi:10.1371/journal.ppat.1000535)
Supplement: Figure S5 — Cell surface localization of HaPrP molecules expressed in CHO cells. CHO cell lines stably expressing wild type and mutant HaPrP were treated with (lanes 3 and 4) or without (lanes 1 and 2) PI-PLC in order to assess anchorage to the outer leaflet of the plasma membrane via the GPI anchor. Following treatment, proteins in the PIPLC incubation media (Media, lanes 1 and 3) were precipitated, and the cells (Lysate, lanes 2 and 4) were harvested in lysis buffer. PrP was detected in the precipitated protein and cell lysate samples by Western blotting. (0.32 MB PDF) [file ppat.1000535.s006.pdf]

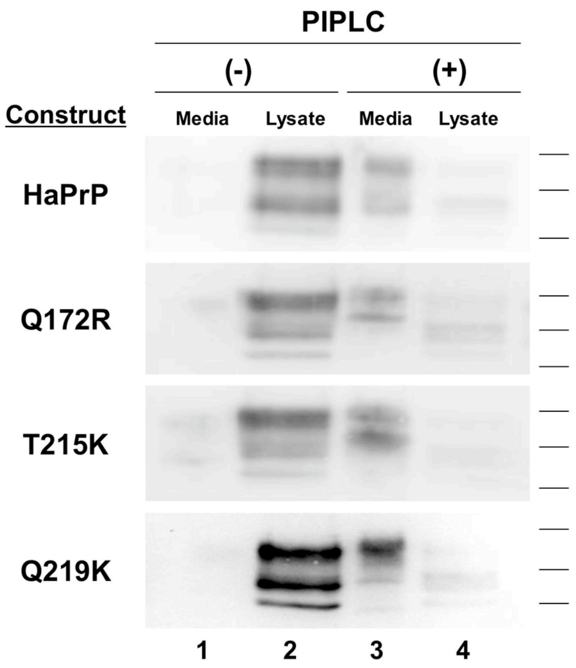

Figure S5.

Cell surface localization of HaPrP molecules expressed in CHO cells.

CHO cell lines stably expressing wild type and mutant HaPrP were treated with (*lanes 3* and *4*) or without (*lanes 1*and *2*) PI-PLC in order to assess anchorage to the outer leaflet of the plasma membrane via the GPI anchor. Following treatment, proteins in the PI-PLC incubation media (*Media*, *lanes 1*and *3*) were precipitated, and the cells (*Lysate*, *lanes 2* and *4*) were harvested in lysis buffer. PrP was detected in the precipitated protein and cell lysate samples by Western blotting.
