# Supplementary material for: Trans-Dominant Inhibition of Prion Propagation In Vitro Is Not Mediated by an Accessory Cofactor
Source: PLoS Pathog. 2009 Jul 31;5(7):e1000535. doi: 10.1371/journal.ppat.1000535 (PMC2713408; doi:10.1371/journal.ppat.1000535)
Supplement: Figure S6 — Detergent solubility of HaPrP molecules expressed in CHO cells. CHO cell lines stably expressing wild type and mutant HaPrP were harvested in lysis buffer containing 0.5% Triton-X 100 and 0.5% DOC. A portion of each cell lysate was removed (TOT, lane 1), the remainder of each lysate was centrifuged at 100,000×g, and the supernatant and pellet fractions were isolated. Equivalent amounts of the Total lysates, supernatant (SUP, lane 2) and pellet (PEL, lane 3) fractions were resuspended in SDS-PAGE loading buffer and rPrP was detected in each sample by Western blotting. (0.22 MB PDF) [file ppat.1000535.s007.pdf]

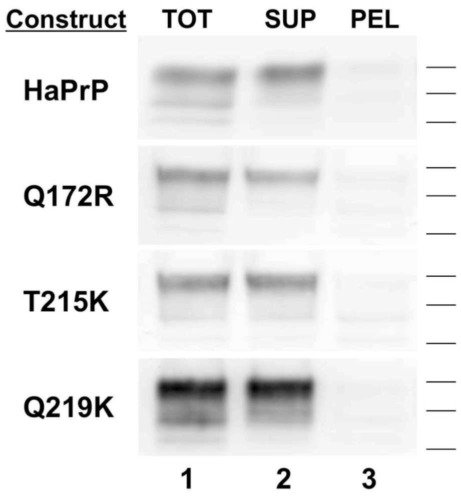

Figure S6.

Detergent solubility of HaPrP molecules expressed in CHO cells.

CHO cell lines stably expressing wild type and mutant HaPrP were harvested in lysis buffer containing 0.5 % Triton-X 100 and 0.5 % DOC. A portion of each cell lysate was removed (*TOT*, *lane 1*), the remainder of each lysate was centrifuged at 100,000 x *g*, and the supernatant and pellet fractions were isolated. Equivalent amounts of the *Total* lysates, supernatant (*SUP*, *lane 2*) and pellet (*PEL*, *lane 3*) fractions were resuspended in SDS-PAGE loading buffer and rPrP was detected in each sample by Western blotting.
